# Genetic variations in *STAT4*,*C2*,*HLA-DRB1* and *HLA-DQ* associated with risk of hepatitis B virus-related liver cirrhosis

**DOI:** 10.1038/srep16278

**Published:** 2015-11-05

**Authors:** De-Ke Jiang, Xiao-Pin Ma, Xiaopan Wu, Lijun Peng, Jianhua Yin, Yunjie Dan, Hui-Xing Huang, Dong-Lin Ding, Lu-Yao Zhang, Zhuqing Shi, Pengyin Zhang, Hongjie Yu, Jielin Sun, S. Lilly Zheng, Guohong Deng, Jianfeng Xu, Ying Liu, Jinsheng Guo, Guangwen Cao, Long Yu

**Affiliations:** 1 State Key Laboratory of Genetic Engineering, Collaborative Innovation Center for Genetics and Development, School of Life Sciences, Fudan University, Shanghai, China; 2 Ministry of Education Key Laboratory of Contemporary Anthropology, School of Life Sciences, Fudan University, Shanghai, China; 3 Center for Genetic Epidemiology, School of Life Sciences, Fudan University, Shanghai, China; 4 Center for Genetic Translational Medicine and Prevention, Fudan University, Shanghai, China; 5 Center for Cancer Genomics, Wake Forest School of Medicine, Winston-Salem, NC, USA; 6 Program for Personalized Cancer Care, NorthShore University HealthSystem, the University of Chicago, IL, USA; 7 National Laboratory of Medical Molecular Biology, Institute of Basic Medical Sciences, Chinese Academy of Medical Sciences, School of Basic Medicine, Peking Union Medical College, Beijing, China; 8 Division of Digestive Diseases, Zhongshan Hospital, Department of Internal Medicine, Shanghai Medical College, Fudan University, Shanghai, China; 9 Department of Epidemiology, Second Military Medical University, Shanghai, China; 10 Department of Infectious Diseases, Southwest Hospital, Institute of Immunology, Third Military Medical University, and Chongqing Key Laboratory of Infectious Diseases, Chongqing, China; 11 Fudan Institute of Urology, Huashan Hospital, Fudan University, Shanghai, China; 12 Institute of Biomedical Science, Fudan University, Shanghai, China

## Abstract

Recent genome-wide associated studies (GWASs) have revealed several common loci associated with the risk of hepatitis B virus (HBV)- or hepatitis C virus (HCV)-related hepatocellular carcinoma (HCC). We selected 15 single nucleotide polymorphisms (SNPs) identified through GWASs on HBV- or HCV-related HCC, and genotyped them in two independent Chinese cohorts of chronic HBV carriers, including 712 LC cases and 2601 controls. The association of each SNP with the risk of HBV-related LC was assessed by meta-analysis of the two cohorts. Of the 12 SNPs reported in HBV-related HCC GWASs, five SNPs (rs7574865 in *STAT4*, rs9267673 near *C2*, rs2647073 and rs3997872 near *HLA-DRB1* and rs9275319 near *HLA-DQ*), were found to be significantly associated with the risk of HBV-related LC (rs7574865: *P* = 1.79 × 10^−2^, OR = 1.17, 95% CI = 1.03–1.34; rs9267673: *P* = 4.91 × 10^−4^, OR = 1.37, 95% CI = 1.15–1.63; rs2647073: *P* = 3.53 × 10^−5^, OR = 1.63, 95% CI = 1.29–2.06; rs3997872: *P* = 4.22 × 10^−4^, OR = 1.86, 95% CI = 1.32–2.62; rs9275319: *P* = 1.30 × 10^−2^, OR = 1.32, 95% CI = 1.06–1.64). However, among the three SNPs associated with the risk of HCV-related HCC in previous GWASs, none of them showed significant association with the risk of HBV-related LC. Our results suggested that genetic variants associated with HBV-related hepatocarcinogenesis may already play an important role in the progression from CHB to LC.

Hepatitis B virus (HBV) infection is one of the most serious and prevalent health problems worldwide, with endemic areas in Sub-Saharan Africa and Southeast Asia, especially China^1^. It was estimated that approximately one-third of the world’s population show serological evidence of current or past HBV infection^1^. Partial of the HBV infected person will develop persistent HBV infection, and the risk of persistence is correlated closely with the patient’s age at the time of infection. Roughly 95% of neonates, 20–30% of children (aged 1–5 years) and less than 5% of adults will develop chronic hepatitis B (CHB) after HBV infection^1^. Although highly effective vaccines against HBV have been available since 1982, there are still more than 350 million CHB carriers worldwide^1^,^2^.

In the course of persistent HBV infection, inflammation forms the pathogenetic basis of CHB that can lead to nodular fibrosis, which can sequentially progress to liver cirrhosis (LC) and, eventually, hepatocellular carcinoma (HCC)^1^,^2^. During a 5-year period, 10–20% of patients with CHB will develop LC^3^. Most cases of HBV-related HCC (70%–80%) occur in patients with HBV-related LC^4^, and the 5-year cumulative incidence of HCC in patients with HBV-related LC is 15% in high endemic areas and 10% in the West^5^. The risk of development from CHB to LC as well as HCC has been attributed to various factors, including viral, environmental and host genetic factors^6^.

During the last several decades, an increasing number of molecular genetic association studies have revealed a number of genetic predispositions associated with risk of HBV-related LC and HCC^7^. With the recent advances in high-density single nucleotide polymorphism (SNP) genotyping arrays and statistical methodology, genome-wide association study (GWAS) has heralded a new era of gene-discovery for complex diseases^8^. To date, there have been several GWASs on HBV-related HCC^9^,^10^,^11^,^12^,^13^, including one GWAS by our group^13^. However, most of these studies focused on HCC at one time point, which might encompass the progression from CHB to LC^14^, and did not address the question of whether these genetic factors are involved in the progress from CHB to HBV-related LC. In addition, two GWASs on hepatitis C virus (HCV)-related HCC have also been carried out and revealed several genetic variants associated with the risk of HCV-related HCC, but they did not report if these variants are also involved in the course of HBV induced diseases^15^,^16^.

In the present study, we investigated the associations of genetic variants discovered by HBV- and HCV-related HCC GWASs with the risk of the progression from CHB to LC in Chinese population.

## Results

Table 1 illustrates the distribution of the values of the main demographic variables of the 3313 participants enrolled in this study. For both of the two cohorts recruited from Shanghai and Beijing, major of the cases and controls were male. The mean age of the cases and controls were 49.53 and 51.00, respectively, in Shanghai cohort, as well as 48.19 and 42.73, respectively, in Beijing cohort.

A total of 17 SNPs, i.e. rs17401966, rs7574865, rs12682266, rs7821974, rs2275959, rs1573266, rs4678680, rs9267673, rs2647073, rs3997872, rs9272105, rs9275319, rs12663434, rs7749730, rs9444730, rs12100561, and rs455804 were selected from five published GWASs on HBV-related HCC, including our own GWAS paper^13^, as well as rs2596542, rs9275572 and rs1012068 selected from two published GWASs on HCV-related HCC^15^,^16^. Among them, rs12682266, rs7821974, rs2275959 and rs1573266 at chromosome 8 as well as rs12663434, rs7749730 and rs9444730 at chromosome 6 were in strong linkage disequilibrium (LD) (data not shown). Thus we only selected rs12682266 and rs7749730 respectively, which were relatively shown more evidence of association with the risk of HBV-related HCC in our GWAS data (Supplementary Table S1), for further analysis.

Characteristic information of the genotyped SNPs regarding chromosome location, related genes, relative distances to genes, base changes, risk alleles as well as risk allele frequencies in Chinese Han in Beijing from HapMap are presented in Table 2. All the SNPs located in non-protein-coding regions or even outside of genes with minor allele frequency (MAF) >0.05 in Chinese Han population except for rs4678680 (MAF = 0.037) and rs3997872 (MAF = 0.037). All the SNPs were in Hardy-Weinberg equilibrium (HWE) (*P* > 0.05) both in cases and controls among Shanghai and Beijing populations (Supplementary Table S2).

The results of the association study for each SNP selected from HBV-related HCC susceptibility GWAS with the risk of progression from CHB to LC are shown in Table 3. Of the 12 SNPs analyzed, five SNPs, i.e., rs7574865 in signal transducer and activator of transcription 4 (*STAT4*), rs9267673 near complement component 2 (*C2*), rs2647073 and rs3997872 near human leukocyte antigen (*HLA)-DRB1* and rs9275319 near *HLA-DQ* were significantly associated with the risk of progression from CHB to LC (rs7574865: *P* = 1.79 × 10^−2^, OR = 1.17, 95% CI = 1.03–1.34; rs9267673: *P* = 4.91 × 10^−4^, OR = 1.37, 95% CI = 1.15–1.63; rs2647073: *P* = 3.53 × 10^−5^, OR = 1.63, 95% CI = 1.29–2.06; rs3997872: *P* = 4.22 × 10^−4^, OR = 1.86, 95% CI = 1.32–2.62; rs9275319: *P* = 1.30 × 10^−2^, OR = 1.32, 95% CI = 1.06–1.64). After false discovery rate (FDR) correction for multiple testing, associations between the five SNPs and risk of LC were still significant (*P* < 0.05). As for the other SNPs, no significant differences of allele frequencies were identified between the case and control groups. We found no evidence for heterogeneity of ORs for these 12 SNPs among these two populations (test for heterogeneity *P* > 0.05 for all SNPs).

Interestingly, at three of the five significant SNPs, i.e., rs7574865, rs9267673 and rs9275319, the directions of the associations were consistent with those from the analyses of the risk of HBV-related HCC in our previous GWAS data (Supplementary Table S1), although rs9267673 didn’t reach the significant level. Unfortunately, we were unable to analyze the association of the other two SNPs with the risk of HBV-related HCC for lack of their allele frequencies in our GWAS data. Similarly, rs9272105 significantly associated with the risk of HBV-related HCC in our GWAS data also showed the same direction of effect in the progression from CHB to LC, though its association with the risk of HBV-related LC didn’t reach significant level (Table 3).

We further examined the associations between SNPs selected from HCV-related HCC susceptibility GWAS and the risk of HBV-related LC. No evidence of significant associations for any of these SNPs was observed. Nevertheless, it’s worth noting that one SNP near *HLA-DQA2* (rs9275572) showing a significant association (*P* = 0.02, OR = 0.84, 95% CI = 1.32–2.62) with the risk of HBV-related HCC (Supplementary Table S1) exhibited an opposite direction of effect in the progression from CHB to LC (*P* = 0.6, OR = 1.04, 95% CI = 0.90-1.20) (Table 4). We did not observe evidence for heterogeneity between studies at any of the three loci (test for heterogeneity *P * > 0.05 for all SNPs).

**Figure 1:**
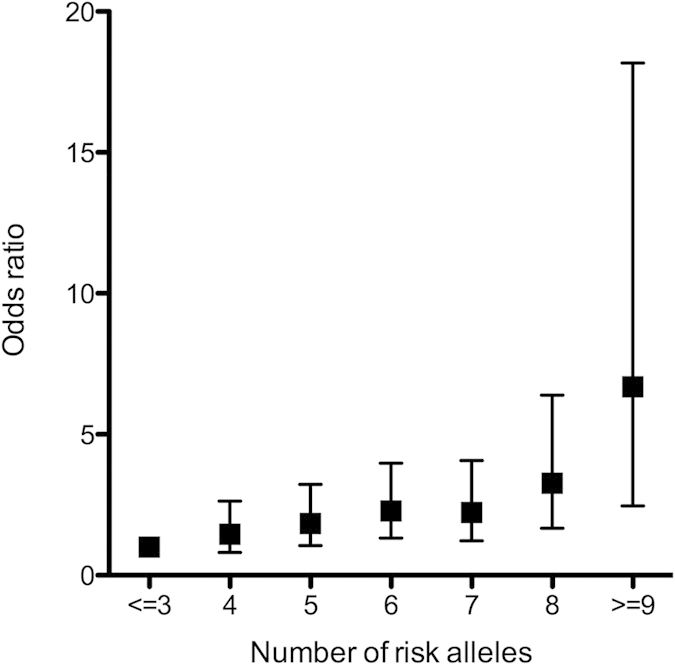
Plot of the increasing crude ORs for hepatitis B virus-related liver cirrhosis with increasing number of risk alleles. The vertical bars represent the 95% confidence intervals.

In order to assess if subjects with more risk alleles of the five LC risk-associated SNPs are more likely to develop LC, we performed cumulative effect analysis. We found a gradual increase in OR with a greater number of hazard alleles. That is, compared with individuals carrying less than or equal to 3 risk alleles, individuals carrying 4, 5, 6, 7, 8, and more than or equal to 9 risk alleles had an adjusted OR of 1.46 (95% CI, 0.81–2.63, *P* = 0.21), 1.84 (95% CI, 1.05–3.22, *P* = 0.03), 2.29 (95% CI, 1.32–3.97, *P* = 3.39 × 10^−3^), 2.23 (95% CI, 1.22–4.08, *P* = 9.32 × 10^−3^), 3.27 (95% CI, 1.67–6.39, *P* = 5.40 × 10^−4^), 6.69 (95% CI, 2.46–18.17, *P* = 1.91 × 10^−4^), respectively, *P*_trend_ = 2.57 × 10^−6^ (Fig. 1).

## Discussion

Chronic infection with the HBV is associated with an increased risk of LC and HCC. Host genetic factors are widely viewed as the common basis of the different outcomes of HBV infection^17^,^18^. In the present study, we examined the association of fifteen SNPs derived from seven GWASs on susceptibility of HBV- or HCV-related HCC with the risk of HBV-related LC in the Chinese population, and found that rs7574865 in *STAT4*, rs9267673 near *C2*, rs2647073 and rs3997872 near *HLA-DRB1* and rs9275319 near *HLA-DQ*, were significantly associated with the risk of HBV-related LC.

LC is an increasing cause of morbidity and mortality, responsible for more than one million deaths every year^4^,^19^. In developed countries, HCV, alcohol misuse and nonalcoholic liver disease are the most common cause of LC; whereas the leading causes in sub-Saharan Africa and most parts of Asia is HBV^4^,^5^. To date, several molecular genetic studies have implicated a number of genetic variants in hepatitis-related LC including two GWASs, each of which revealed two independent susceptibility loci for LC^20^,^21^. However, both of the two GWASs were performed in LC patients developed from chronic hepatitis C. Till present, no GWAS has been conducted to systematically identify genetic variants associated with HBV-related LC. Only one candidate gene-based association study demonstrated a statistical association of rs430397, located in the intron 5 of glucose-regulated protein 78 (*GRP78*), with HBV-related LC^22^.

In our study, rs7574865, which was located in the third intron of *STAT4*, was significantly associated with the risk of progression to LCs in patients with CHB. STAT4 is a transcription factor belonging to STAT family^23^, important members of Janus Kinase (JAK)-STAT pathway, that is required for the development of Th1 cells from naïve CD4^+^ T cells^24^ and interferon-γ (IFN-γ) production in response to interleukin-12 (IL12)^25^ and type I interferon (IFN-α or IFN-β)^26^. In addition to the association of this gene with HBV-related LC and HCC in our own studies, it has been reported that genetic polymorphisms of this gene, especially the rs7574865, are also associated with numerous autoimmune diseases^27^,^28^, indicating a crucial role in the immune system. Interestingly, during fibrosis progression, immune cells secret a variety of growth factors and inflammatory cytokines including IL-6, IFN-γ, IFN-α/β, and IL-22, which have been shown to play key roles in regulating liver fibrogenesis resulting in collagen deposition and the disruption of the normal liver architecture^29^,^30^. In addition, rs7574865 has been shown to influence the mRNA level of STAT4 in our published HBV-related HCC GWAS^13^. These previous findings enhance the biological plausibility that this SNP in *STAT4* may play an important role in the liver fibrosis progression, although the molecular mechanism remains uncertain and further functional studies are required.

The rest of four HBV-related LC risk-related SNPs (rs9267673, rs264707, rs3997872 and rs9275319) were all located in molecules that belong to the HLA class II, which has been considered to be the most important host factors with respect to outcomes of HBV and HCV infections. Among them, rs9267673, rs2647073 and rs3997872 were already shown to be associated with LC in the initial GWAS study^9^. HLA system is the most important region in the human genome related to infection, inflammation, autoimmunity, and transplantation medicine^31^. Interactions among HLA-restricted T lymphocytes, B lymphocytes, natural killer cells, and cytokines influence immune response to viral infection. Increasing numbers of case-control association studies and GWASs have demonstrated that several variants in HLA class II region were associated with persistent HBV infection as well as HCC progression in patients with HBV^32^,^33^,^34^. Given the significant association between HLA region and outcome of HBV infection in previous studies, together with our robust statistic results, it is natural to speculate that polymorphisms in such region may also associated with the progression to LC in patients with CHB.

Unfortunately, we observed no significant evidence of association between SNPs identified by HCV-related HCC GWAS and the risk of HBV-related LC, which also failed to be replicated in our own HBV-related HCC GWAS population. The main reason for this result might be the difference between these two viruses. Although there is certain similarity in clinical manifestations of hepatitis induced by these viruses and creating background for subsequent development for LC and HCC, their molecular organization, replication strategy and functions of constituent proteins are different. In addition, HCV increases the risk for HCC by inducing fibrosis and, eventually, cirrhosis; while HBV can cause HCC in the absence of cirrhosis. Thus, different mechanisms of liver fibrogenesis and carcinogenesis might operate in HBV- and HCV-related chronic inflammation.

In summary, we explored the association of GWAS-identified HBV- and HCV-related susceptibility SNPs with the progression from CHB to LC in two independent cohorts of Chinese population. Our study confirms that five of these SNPs (rs7574865 in *STAT4*, rs9267673 near *C2*, rs2647073 and rs3997872 near *HLA-DRB1* and rs9275319 near *HLA-DQ*) also affect individual susceptibility to HBV-related LC. Thus, these findings provide new insights into the etiology of LC.

## Materials and Methods

### Study population

In this study, two independent case-control cohorts including 440 HBV-related LC cases and 1265 HBV positive controls from eastern China (Shanghai) as well as 272 cases and 1336 controls from northern China (Beijing) were recruited (Table 1). All participants were genetically unrelated ethnic Han Chinese. All the LC patients were diagnosed by pathologic exams, laboratory features, and the findings of computed tomography (CT) or ultrasonography based on the following criteria: thrombocytopenia (<150,000 platelets per μL), cirrhotic configuration of the liver (nodular liver surface or caudate lobe hypertrophy) and/or splenomegaly confirmed in imaging studies, or the presence of varices (abnormally enlarged veins, detected by upper endoscopy or cross-sectional images). Both of cases and controls were CHB carriers that were positive for both HBV surface antigen and immunoglobulin G antibody to HBV core antigen for at least 6 months. All subjects in this study were negative for antibodies to HCV, hepatitis D virus, or human immunodeficiency virus; and had no other types of liver disease, such as autoimmune hepatitis, toxic hepatitis, alcoholism-related cirrhosis and HCC. The study was performed in accordance with approved guidelines and was approved by the Department of Scientific Research of Fudan University and local ethical committees from all the participating centers. An informed consent to participate in the study was obtained from each subject, in accordance with the declaration of Helsinki principles. All study participants approved the storage of their frozen DNA specimens, for research purposes, in our laboratory.

### SNP selection and genotyping

We used the NHGRI GWAS Catalog (http://www.genome.gov/26525384) to search for SNPs identified by the published HBV- and HCV-related HCC susceptibility GWAS. Additionally, we searched PubMed for reports of HBV- and HCV-related HCC susceptibility loci published in English before November 20, 2014. We used a combination of the search terms “genome wide association study” and “hepatitis B virus” or “hepatitis C virus” and “hepatocellular carcinoma”. We identified five HBV-related HCC GWASs and two HCV-related HCC GWASs, which revealed 12 independent HBV-related HCC susceptibility loci and 3 HCV-related HCC susceptibility loci. Information of these SNPs were extracted from original articles or searched in the SNP database on National Center for Biotechnology Information.

Genotyping analysis for all SNPs were conducted using the Sequenom MassARRAY multiplex genotyping platform (Sequenom) at the Fudan-VARI laboratory and TaqMan assays at the State Key Laboratory of Genetic Engineering, Fudan University, according to the manufacturers’ instructions. Primers and TaqMan MGB probes were purchased from Life Technologies (Foster City, CA). Several quality-control measures implemented in genotyping analysis are as follows: duplicate test samples were mixed in the plates; two water samples (PCR negative controls) were included in each 96-well plate; and persons performing the genotyping assays were blinded to the identity of the samples. The concordance rates for quality-control samples were >99% for all assays.

### Statistical analysis

Continuous variables were presented as mean ± standard deviation while categorical variables were expressed as frequencies (%). HWE tests of all the genotyped SNPs in cases, controls and all samples were performed using chi-square test. LD was assessed using Haploview 4.2 software by determining D’ and r^2^ values. The associations of SNPs with the risk of progression from CHB to LC or HBV-related HCC were estimated by computing the odds ratios (ORs) and their 95% confidence intervals (CIs) from logistic regression analysis under an additive model adjusting for gender and age using PLINK (v1.07). A Cochrane chi-square-based Q-test was performed to test the heterogeneity among studies or cohorts. Meta-analysis was performed based on the association results using the fixed-effects model (Mantel-Haenszel model) if the result of the heterogeneity test showed *P*_het_ ≥ 0.05 or using the random-effects model (DerSimonian and Laird model) if *P*_het_ < 0.05. We performed FDR correction for multiple testing using R software. The cutoff for significant association after FDR correction for multiple testing was set at *P* value <0.05. All statistical tests were two sided. We also tested the cumulative effects of the five significant SNPs by counting the number of risk alleles associated with LC risk in each subject. The OR for those carrying any combination of 4, 5, 6, 7, 8 or more than or equal to 9 risk alleles was estimated by comparing them with those carrying less than or equal to 3 risk alleles with the logistic regression analysis adjusting age and gender.

## Additional Information

**How to cite this article**: Jiang, D.-K. *et al.* Genetic variations in *STAT4, C2, HLA-DRB1* and *HLA-DQ* associated with risk of hepatitis B virus-related liver cirrhosis. *Sci. Rep.* **5**, 16278; doi: 10.1038/srep16278 (2015).

## Supplementary Material

Supplementary Table S1 & S2

## Figures and Tables

**Table 1:** Demographic characteristics of subjects analyzed in the study.

Subjects	Number	Gender, n (%)		Age, years		
		Female	Male	Mean (SD)	≤50, n (%)	>50, n (%)
Shanghai cohort^a^						
Cases	440	109 (24.77)	331 (75.23)	49.53 (10.94)	249 (56.59)	191 (43.41)
Controls	1265	464 (36.68)	801 (63.32)	51.00 (12.89)	536 (42.47)	729 (57.63)
Beijing cohort^b^						
Cases	272	65 (23.90)	207 (76.10)	48.19 (12.30)	160 (58.82)	112 (41.18)
Controls	1336	491 (36.75)	845 (63.25)	42.73 (15.04)	947 (70.88)	389 (29.12)

^a^Recruited from Shanghai in eastern China.

^b^Recruited from Beijing in northern China.

**Table 2:** Information of the SNPs previously reported to be associated with the risk of HBV- and HCV-related HCC by GWASs.

Chr.	Related gene	Distance to gene	Position	SNP	Allele^a^	Risk allele^b^	RAF in CHB	Ref.
1	*KIF1B*	Intron 24	10308058	rs17401966	G/A	A	0.733	10
2	*STAT4*	Intron 3	191672878	rs7574865	T/G	G	0.650	13
3	*GLB1*	18 kb upstream	32995039	rs4678680	G/T	G	0.037	9
6	*C2*	12 kb upstream	31991658	rs9267673	T/C	T	0.159	9
6	*HLA-DRB1*	16 kb downstream	32681992	rs2647073	C/A	C	0.098	9
6	*HLA-DRB1*	23 kb downstream	32688595	rs3997872	A/T	A	0.037	9
6	*HLA-DQA1/DRB1*	96kb downstream of HLA-DQA1	32707977	rs9272105	G/A	A	0.524	12
6	*HLA-DQ*	42 kb downstream of HLA-DQB2	32774273	rs9275319	G/A	A	0.783^c^	13
6	*BACH2*	Intron 5	90791705	rs7749730	G/A	A	0.890	9
8	—	—	37548149	rs12682266	A/G	G	0.488	11
14	*C14orf143*	Intron 5	89370788	rs12100561	A/G	A	0.366	9
21	*GRIK1*	Intron 1	30068040	rs455804	A/C	C	0.683	12
6	*MICA*	4.7 kb upstream	31474574	rs2596542	T/C	T	0.256	15
6	*HLA-DQA2*	Intron 1	32786977	rs9275572	A/G	A	0.317	15
22	*DEPDC5*	Intron 32	30595903	rs1012068	G/T	G	0.134	16

Chr. chromosome; RAF, risk allele frequency; CHB, Chinese Han in Beijing from in HapMap.

^a^Minor allele/major allele.

^b^Risk allele means the allele associated with increased risk of HBV-related HCC in the original GWAS.

^C^Allele frequency in CHB+JPT in HapMap.

**Table 3:** Results of association between SNPs previously reported by HBV-related HCC GWAS and the risk of progression from CHB to LC.

Gene	SNP	Allele^a^	RAF^b^ of Shanghai cohort		RAF^b^ of Beijing cohort		OR_meta_ (95% CI)^c^,^d^	*P*_meta_^c^	*P*_meta_-Adjusted^e^	*P*_het._
			Cases	Controls	Cases	Controls				
*KIF1B*	rs17401966	G/A	0.33	0.29	0.28	0.28	1.13 (0.99–1.30)	8.03 × 10^−2^	1.64 × 10^−1^	0.522
*STAT4*	rs7574865	T/G	0.72	0.67	0.72	0.69	1.17 (1.03–1.34)	1.79 × 10^−2^	4.18 × 10^−2^	0.894
*GLB1*	rs4678680	G/T	0.94	0.93	0.92	0.93	1.13 (0.87–1.47)	3.45 × 10^−1^	4.57 × 10^−1^	0.958
*C2*	rs9267673	T/C	0.17	0.12	0.13	0.12	1.37 (1.15–1.63)	4.91 × 10^−4^	1.74 × 10^−3^	0.680
*HLA-DRB1*	rs2647073	C/A	0.09	0.05	0.10	0.08	1.63 (1.29–2.06)	3.53 × 10^−5^	3.75 × 10^−4^	0.886
*HLA-DRB1*	rs3997872	A/T	0.97	0.94	0.97	0.95	1.86 (1.32–2.62)	4.22 × 10^−4^	1.66 × 10^−3^	0.997
*HLA-DQA1/DRB1*	rs9272105	A/G	0.43	0.41	0.42	0.39	1.07 (0.95–1.20)	2.59 × 10^−1^	3.87 × 10^−1^	0.981
*HLA-DQ*	rs9275319	G/A	0.91	0.89	0.93	0.90	1.32 (1.06–1.64)	1.30 × 10^−2^	3.36 × 10^−2^	0.989
*BACH2*	rs7749730	G/A	0.17	0.15	0.15	0.17	1.06 (0.89–1.26)	5.14 × 10^−1^	5.56 × 10^−1^	0.387
*–*	rs12682266	A/G	0.50	0.50	0.47	0.46	1.01 (0.89–1.14)	8.97 × 10^−1^	6.86 × 10^−1^	1.000
*C14orf143*	rs12100561	A/G	0.41	0.40	0.39	0.41	1.00 (0.88–1.14)	9.42 × 10^−1^	6.96 × 10^−1^	0.952
*GRIK1*	rs455804	A/C	0.33	0.32	0.29	0.32	1.00 (0.87–1.15)	9.88 × 10^−1^	7.06 × 10^−1^	0.831

RAF, risk allele frequency; OR, odds ratio; CI, confidence interval.

^a^Minor allele/major allele.

^b^RAF means the allele frequency of cases is lower than that of controls in the Shanghai cohort or both of the two cohorts.

^c^The data was analyzed by meta-analysis using the fixed-effects model (the Mantel-Haenszel model) based on the association results, which were generated by logistic regression analysis under an additive model adjusting for gender and age in the two cohorts.

^d^The ORs and 95%CIs were calculated by considering the non-risk allele as a reference.

^e^False discovery rate (FDR) correction for multiple testing.

**Table 4:** Results of association between SNPs previously reported by HCV-related HCC GWAS and the risk of progression from CHB to LC.

Gene	SNP	Allele^a^	RAF^b^ of Shanghai cohort		RAF^b^ of Beijing cohort		OR_meta_ (95%CI)^c^,^d^	*P*_meta_^c^	*P*_meta_-Adjusted^e^	*P*_het._
			Cases	Controls	Cases	Controls				
*MICA*	rs2596542	T/C	0.30	0.28	0.29	0.25	1.10 (0.96–1.26)	1.65 × 10^−1^	2.87 × 10^−1^	0.999
*HLA-DQA2*	rs9275572	A/G	0.24	0.24	0.26	0.24	1.04 (0.90–1.20)	6.14 × 10^−1^	5.99 × 10^−1^	0.998
*DEPDC5*	rs1012068	G/T	0.25	0.24	0.24	0.24	0.99 (0.85–1.14)	8.64 × 10^−1^	6.78 × 10^−1^	0.945

RAF, risk allele frequency; OR, odds ratio; CI, confidence interval.

^a^Minor allele/major allele.

^b^RAF means the allele frequency of cases is lower than that of controls in the Shanghai cohort or both of the two cohorts.

^c^The data was analyzed by meta-analysis using the fixed-effects model (the Mantel-Haenszel model) based on the association results, which were generated by logistic regression analysis under an additive model adjusting for gender and age in the two cohorts.

^d^The ORs and 95%CIs were calculated by considering the non-risk allele as a reference.

^e^False discovery rate (FDR) correction for multiple testing.
