# Supplementary material for: Genetic variations in STAT4,C2,HLA-DRB1 and HLA-DQ associated with risk of hepatitis B virus-related liver cirrhosis
Source: Sci Rep. 2015 Nov 5;5:16278. doi: 10.1038/srep16278 (PMC4633722; doi:10.1038/srep16278)
Supplement: Supplementary Table S1 & S2 [file srep16278-s1.doc]

**Genetic variations in *STAT4*, *C2*, *HLA-DRB1* and *HLA-DQ* associated with risk of hepatitis B virus-related liver cirrhosis**

De-Ke Jiang^1-5*†^, Xiao-Pin Ma^1*^, Xiaopan Wu^6^, Lijun Peng^7^, Jianhua Yin^8^, Yunjie Dan^9^, Hui-Xing Huang^1^, Dong-Lin Ding^1^, Lu-Yao Zhang^1^, Zhuqing Shi^1-4^, Pengyin Zhang^1-4^, Hongjie Yu^1-4^, Jielin Sun^5^, S. Lilly Zheng^5^, Guohong Deng^9^, Jianfeng Xu^1-5,10,11^, Ying Liu^6^, Jinsheng Guo^7†^, Guangwen Cao^8†^ and Long Yu^1,12^

^1^State Key Laboratory of Genetic Engineering, Collaborative Innovation Center for Genetics and Development, School of Life Sciences, Fudan University, Shanghai, China;

^2^Ministry of Education Key Laboratory of Contemporary Anthropology, School of Life Sciences, Fudan University, Shanghai, China;

^3^Center for Genetic Epidemiology, School of Life Sciences, Fudan University, Shanghai, China;

^4^Center for Genetic Translational Medicine and Prevention, Fudan University, Shanghai, China;

^5^Center for Cancer Genomics, Wake Forest School of Medicine, Winston-Salem, NC, USA;

^6^Program for Personalized Cancer Care, NorthShore University HealthSystem, the University of Chicago, IL, USA;

^7^National Laboratory of Medical Molecular Biology, Institute of Basic Medical Sciences, Chinese Academy of Medical Sciences, School of Basic Medicine, Peking Union Medical College, Beijing, China;

^8^Division of Digestive Diseases, Zhongshan Hospital, Department of Internal Medicine, Shanghai Medical College, Fudan University, Shanghai, China;

^9^Department of Epidemiology, Second Military Medical University, Shanghai, China;

^10^Department of Infectious Diseases, Southwest Hospital, Institute of Immunology, Third Military Medical University, and Chongqing Key Laboratory of Infectious Diseases, Chongqing, China;

^11^Fudan Institute of Urology, Huashan Hospital, Fudan University, Shanghai, China;

^12^Institute of Biomedical Science, Fudan University, Shanghai, China.

^*^These authors contributed equally to this work.

**^†^Corresponding Authors:**

Deke Jiang, PhD

State Key Laboratory of Genetic Engineering, School of Life Sciences, Fudan University, 2005 Songhu Rd., Shanghai 200438, China.

Phone: +86-21-51630617

Fax: +86-21-51630618

Email: dekejiang@fudan.edu.cn

Guangwen Cao, MD, PhD

Department of Epidemiology, Second Military Medical University, Shanghai, China

Phone: +86-21-81871060

Fax: +86-21-81871060

Email: gcao@smmu.edu.cn

Jinsheng Guo, MD, PhD

Division of Digestive Diseases, Zhongshan Hospital, Department of Internal Medicine, Shanghai Medical College, Fudan University, 180 Fenglin Rd., Shanghai 200032, China.

Phone: +86-21-64041990x2424

Fax: +86-21-64038472

Email: guo.jinsheng@zs-hospital.sh.cn

**Supplementary Table S1**. The SNPs previously reported to be associated with the risk of HBV and HCV-related HCC by GWAS and association of these SNPs with HBV-related HCC risk in our GWAS data

| Gene | SNP | Position | Allele^a^ | OR (95%CI)^b^ | *P* | In our GWAS data | | | |
| --- | --- | --- | --- | --- | --- | --- | --- | --- | --- |
|  |  |  |  |  |  | F_A | F_U | OR (95%CI)^b^ | *P* |
| *KIF1B* | rs17401966 | 10308058 | G/A | 0.61 (0.55–0.67) | 1.70E-18 | 0.27 | 0.28 | 0.98 (0.87–1.11) | 0.79 |
| *STAT4* | rs7574865 | 191672878 | T/G | 1.21 (1.14–1.28) | 2.48E–10 | 0.72 | 0.67 | 1.27 (1.12–1.44) | 1.53E-4 |
| *-* | rs12682266 | 37548149 | A/G | 0.72 (NR) | 3.76E-5 | 0.47 | 0.49 | 0.92 (0.82–1.03) | 0.14 |
| *-* | rs7821974 | 37569159 | C/T | 0.75 (NR) | 2.32E-4 | 0.49 | 0.5 | 0.95 (0.85–1.06) | 0.36 |
| *-* | rs2275959 | 37574217 | A/G | 1.31 (NR) | 5.19E-4 | 0.46 | 0.45 | 1.03 (0.92–1.16) | 0.61 |
| *-* | rs1573266 | 37581577 | A/G | 0.72 (NR) | 2.71E-5 | 0.4 | 0.4 | 0.98 (0.85–1.11) | 0.71 |
| *GLB1* | rs4678680 | 32995039 | G/T | 2.27 (1.68–3.08) | 2.00E-7 | 0.07 | 0.07 | 1.10 (0.89–1.37) | 0.37 |
| *C2* | rs9267673 | 31991658 | T/C | 1.97 (1.47–2.64) | 2.00E-6 | 0.1 | 0.09 | 1.14 (0.94–1.38) | 0.17 |
| *HLA-DRB1* | rs2647073 | 32681992 | C/A | 1.94 (1.40–2.69) | 6.00E-5 | NA | NA | NA | NA |
| *HLA-DRB1* | rs3997872 | 32688595 | A/T | 0.47 (0.33–0.67) | 2.00E-5 | NA | NA | NA | NA |
| *HLA-DQA1/DRB1* | rs9272105 | 32707977 | A/G | 1.28 (1.22–1.35) | 5.24E-22 | 0.52 | 0.49 | 1.20 (1.08-1.35) | 1.21E-3 |
| *HLA-DQ* | rs9275319 | 32774273 | G/A | 1.49 (1.36–1.63) | 2.72E–17 | 0.92 | 0.88 | 1.60 (1.32–1.93) | 1.11E-6 |
| *BACH2* | rs12663434 | 90790803 | A/C | 0.60 (0.48–0.77) | 3.00E-5 | 0.17 | 0.17 | 1.02 (0.88–1.18) | 0.79 |
| *BACH2* | rs7749730 | 90791705 | G/A | 0.59 (0.46–0.75) | 1.00E-5 | 0.17 | 0.17 | 1.02 (0.88–1.19) | 0.76 |
| *BACH2* | rs9444730 | 90790058 | G/T | 0.59 (0.46–0.75) | 1.00E-5 | 0.17 | 0.16 | 1.02 (0.87–1.18) | 0.85 |
| *C14orf143* | rs12100561 | 89370788 | A/G | 1.52 (1.26–1.83) | 4.00E-6 | 0.42 | 0.43 | 0.99 (0.88–1.10) | 0.80 |
| *GRIK1* | rs455804 | 30068040 | A/C | 0.84 (0.80–0.89) | 5.24E-10 | 0.33 | 0.32 | 1.08 (0.95-1.21) | 0.23 |
| *MICA* | rs2596542 | 31474574 | T/C | 1.39 (1.27–1.52) | 4.21E-13 | 0.25 | 0.27 | 0.91 (0.80–1.04) | 0.16 |
| *HLA-DQA2* | rs9275572 | 32786977 | A/G | 1.30 (1.19–1.42) | 5.97E-9 | 0.18 | 0.21 | 0.84 (0.73–0.97) | 0.02 |
| *DEPDC5* | rs1012068 | 30595903 | *G/T* | 1.75 (1.51–2.03) | 1.27E-13 | 0.23 | 0.21 | 1.14 (0.99–1.30) | 0.06 |

OR, odds ratio; CI, confidence interval; Ref., reference; F_A, frequency of minor allele of affected individuals (CHB patients with HCC); F_U, frequency of minor allele of unaffected individuals (CHB patients without HCC); NA, not available.

^a^Minor allele/major allele.

^b^The ORs and 95%CIs derived from logistic regression analysis under an additive model by considering the major allele as a reference.

**Supplementary Table S2**. Hardy-Weinberg equilibrium test of all the SNPs both in cases and controls among Shanghai and Beijing cohorts

| Chr. | Gene | SNP | Allele^a^ | Proportion of three genotypes in Shanghai cohort (%)^b^ | |  | *P* value of HWE in Shanghai cohort | | |  | Proportion of three genotypes in Beijing cohort (%)^b^ | |  | *P* value of HWE in Beijing cohort | | |
| --- | --- | --- | --- | --- | --- | --- | --- | --- | --- | --- | --- | --- | --- | --- | --- | --- |
|  |  |  |  | Cases | Controls |  | Cases | Controls | All |  | Cases | Controls |  | Cases | Controls | All |
| 1 | *KIF1B* | rs17401966 | G/A | 10/47/43 | 8/41/51 |  | 0.24 | 0.95 | 0.52 |  | 10/36/54 | 8/40/52 |  | 0.13 | 0.72 | 0.25 |
| 2 | *STAT4* | rs7574865 | T/G | 8/40/52 | 11/43/46 |  | 0.81 | 0.56 | 0.50 |  | 8/40/52 | 10/40/50 |  | 1.00 | 0.38 | 0.45 |
| 3 | *GLB1* | rs4678680 | G/T | 1/10/89 | 0/14/86 |  | 0.16 | 0.20 | 0.70 |  | 0/15/85 | 1/13/86 |  | 1.00 | 0.41 | 0.65 |
| 6 | *C2* | rs9267673 | T/C | 4/26/70 | 2/21/77 |  | 0.67 | 0.13 | 0.13 |  | 2/22/76 | 1/20/79 |  | 0.79 | 1.00 | 1.00 |
| 6 | *HLA-DRB1* | rs2647073 | C/A | 2/13/85 | 0/10/90 |  | 1.00 | 0.11 | 0.13 |  | 1/18/81 | 2/12/87 |  | 0.73 | 0.19 | 0.21 |
| 6 | *HLA-DRB1* | rs3997872 | A/T | 0/6/94 | 0/11/89 |  | 1.00 | 1.00 | 1.00 |  | 0/6/94 | 0/10/90 |  | 0.25 | 0.25 | 0.72 |
| 6 | *HLA-DQA1/DRB1* | rs9272105 | A/G | 6/31/63 | 4/31/65 |  | 0.05 | 0.47 | 0.08 |  | 5/32/62 | 5/32/64 |  | 0.48 | 0.43 | 0.30 |
| 6 | *HLA-DQ* | rs9275319 | G/A | 0/17/83 | 1/19/80 |  | 0.56 | 0.31 | 0.60 |  | 0/13/87 | 1/16/82 |  | 1.00 | 0.20 | 0.23 |
| 6 | *BACH2* | rs7749730 | G/A | 4/27/70 | 2/26/72 |  | 0.24 | 0.09 | 0.45 |  | 2/26/72 | 3/28/69 |  | 1.00 | 0.89 | 0.82 |
| 8 | *-* | rs12682266 | A/G | 26/47/26 | 25/49/26 |  | 0.25 | 0.57 | 0.29 |  | 23/47/30 | 21/50/28 |  | 0.40 | 0.82 | 0.85 |
| 14 | *C14orf143* | rs12100561 | A/G | 15/53/33 | 16/49/35 |  | 0.09 | 0.56 | 0.16 |  | 15/49/36 | 18/47/35 |  | 0.70 | 0.33 | 0.52 |
| 21 | *GRIK1* | rs455804 | A/C | 10/47/44 | 11/43/47 |  | 0.28 | 0.47 | 1.00 |  | 9/41/50 | 10/44/46 |  | 0.77 | 1.00 | 0.94 |
| 6 | *MICA* | rs2596542 | T/C | 9/41/50 | 8/38/53 |  | 0.65 | 0.18 | 0.17 |  | 9/38/53 | 7/37/56 |  | 0.30 | 0.38 | 0.16 |
| 6 | *HLA-DQA2* | rs9275572 | A/G | 6/36/58 | 6/34/59 |  | 0.60 | 0.07 | 0.06 |  | 8/36/56 | 6/34/60 |  | 0.43 | 0.22 | 0.12 |
| 22 | *DEPDC5* | rs1012068 | G/T | 6/37/56 | 6/36/58 |  | 1.00 | 0.70 | 0.74 |  | 5/37/58 | 5/37/58 |  | 0.74 | 0.84 | 0.66 |

Chr., chromosome; HWE, Hardy-Weinberg.

^a^Minor allele/major allele.

^b^Minor allele homozygous/heterozygous/major allele homozygous.
